# Developing a Tool for Assessing Perceived Parental Socialization of Emotions in Adolescents and Young Adults

**DOI:** 10.7759/cureus.65748

**Published:** 2024-07-30

**Authors:** Grace S Joyce, Ridhima Shukla

**Affiliations:** 1 Department of Psychology, Christ (Deemed to be University) Delhi NCR, Delhi, IND

**Keywords:** young adults, adolescents, tool construction, exploratory factor analysis, parental socialization of emotion

## Abstract

Introduction: Parenting practices have been described as the practices employed by a parent and the parent-child communication, with the focus on raising a child in the best possible manner while instilling cultural, ethical, and personal values. One similar yet different construct that holds significance in the development of a child is parental socialization of emotion. Parental socialization of emotion is the interaction between the parents and child regarding the emotional experience of the child. This has been considered an essential marker of the development of the emotional and social competence of an individual. There are several scales in the aspect of parenting practices. However, scales for parental socialization of emotion especially considering the perception of adolescents and young adults have yet to fully be explored.

Methods: The aim of the study is to establish a comprehensive tool that enables the measurement of perceived parental socialization of emotion (PPSE). The process of tool construction, data collection, and analysis was done in five phases that included reviewing existing tools and identification of domains (Phase 1); generation of an item pool (Phase 2); content validity, face validity, and inter-rater reliability (Phase 3); finalization of the tool for data collection (Phase 4); and data collection and analysis (Phase 5). The study for tool construction included participants from ages 13-28 years representing adolescents and young adults from schools and colleges located in New Delhi, India, as well as Gwalior, Madhya Pradesh, India. The research design used was a cross-sectional design, and the data were collected through purposive sampling, including males (N = 337) and females (N = 424). Exploratory factor analysis (EFA) was used to check the factorability and for item reduction.

Results: The results aided in reducing the number of items from 160 to 46 including the process of content validity, inter-rater reliability, item-total statistic, and factor loading of EFA. The factors above the eigenvalue of 3 were retained while items above the factor loading of 0.50 were taken into consideration. A good Cronbach’s alpha coefficient of 0.88 for the overall tool was established, with 0.89, 0.84, and 0.87 for the domains Awareness, Acceptance, and Coaching, respectively.

Conclusion: The scale constructed includes both the positive and negative emotions of an individual and tries to understand the perspective of the receiver of the parenting practice. The study helps in understanding the phenomenon of PPSE, which might also aid in creating awareness regarding efficient parenting practices.

## Introduction

Parenting practices have been considered as the interaction between a parent and their child based on an array of values, beliefs, attitudes, and behaviors [1]. One such aspect that includes all the predominant components of parenting practices is the parental socialization of emotions, which has surfaced as an integral dimension, taking into consideration the parent’s role in understanding their child’s expression and regulation of emotions. Parental socialization of emotions aims to address the exchange between a parent and a child in the context of emotions [2,3]. Emphasizing on parenting as an emotional process, an affective model, has been proposed to influence the quality of parenting and its effect on a child’s development [4]. Therefore, parental socialization of emotions can be described as the process of understanding the emotions expressed by the child (Awareness), responding to those emotions (Acceptance), and discussing the emotions expressed by the child (Coaching) [3,5]. Parental socialization of emotions has been famously known to be enhanced by comprehensively explaining the operation, criteria, and path framework of the socialization process with potential outcomes, mediating roles, and causal factors [3]. However, in this study, a different approach inching toward an integration of the domains proposed in parental meta-emotion philosophy and emotion coaching as parenting practice has been taken into consideration. This approach is centered around emotion socialization that influences a child’s outcomes, viewing the entire course as an opportunity to create intimacy and contribute toward the nurturance of the child [2].

The theory of emotion socialization aided in the embarkment of several researchers in the field of parental socialization of emotions with further utilization of the tools created to measure the same (Manual: Katz & Gottman. The meta-emotion interview; 1986). The phenomenon is extensively studied and measured using an array of scales and interviews that elucidate the occurrence and impact on parents and their child. The scales predominantly considered to explore the phenomenon are the Coping with Children's Negative Emotions Scale (CCNES) [6] and the Parent Meta-Emotion Interview (Manual: Katz & Gottman. The meta-emotion interview; 1986). CCNES, although more widely used, is based on the theory proposed by Eisenberg [3]. The scale claims to be based on the theoretical construct given by Eisenberg [3] with the addition of emotional coaching as an element [2] along with the coping and stress theory [7,8]. CCNES includes elements of parental socialization consisting of domains divided into supportive and non-supportive strategies that further have three subdomains, each involving punitive reactions (PR), distress reactions (DR), minimization reactions, expressive encouragement, emotion-focused reactions (EFR), and problem-focused reactions (PFR). The scale comprises various scenarios to which the responder is required to choose from a seven-point scale. The scale having 72 items in total is based on the parental socialization theory by Eisenberg, which suggests the mechanism of parental socialization of emotions as emotion-related socialization behavior (ERBS) with three specific elements: a) parent’s reaction to their child’s emotions, b) discussion of the emotions expressed, and c) parent’s reciprocal expression of the emotions. In view of the theory, the CCNES does not address the three elements of ERBS in a clear manner, thus making it difficult to derive a clear interpretation from the scale.

The earliest scale for parental socialization of emotions developed was named the Parent Meta-Emotion Interview (PMEI) (Manual: Katz & Gottman. The meta-emotion interview; 1986), which was later revised in 2008. This Interview aims to know the manner in which parents perceive their emotions and their child’s emotions. The Interview is based on a coding system that particularly addresses three negative emotions, namely, sadness, anger, and fear, as subscales that are separate for the parent and their child. The subscale for parent reports includes awareness, expressivity, acceptance, parent remediation, and parent regulation. However, the child’s subscale is slightly different and includes child awareness, child acceptance, child coaching, child behavioral strategies, and child dysregulation. The interview measure aids in capturing parental socialization of emotions as a parenting practice, but the scale is lengthy, requiring extensive knowledge of a coding method. The Emotion-Related Parenting Styles Self-Test [9] was introduced, which might be more convenient for exploring parenting practices around emotion socialization; however, a detour was taken to cover parenting styles related to emotions, i.e., emotion coaching, laissez-faire, dismissing, and disapproving.

Both the PMEI and CCNES make it difficult to portray the phenomenon of parental socialization of emotions in an understandable and compact fashion. Moreover, the scales focus on negative emotions, indicating that negative emotions beckon more parental attention as compared to the manifestation of positive emotions. Furthermore, the scales were originally parent self-report scales aimed at assessing parental socialization of emotion styles and practices; however, parent self-reports are susceptible to biases in the perception of parenting practices, which they might claim to implement than the ones that are actually implemented. In addition, parents' understanding of their parenting practices and their experience might differ from their child’s experience. Moreover, parents might be prone to giving socially desirable answers in order to seem rather acceptable [10]. Hence, multiple methods to measure the phenomenon could be used, which could also involve youth self-reports in addition to the observation of parent-child interactions and longitudinal studies.

A youth self-report scale to explore parenting practices could contribute deeply to studies and make them empirically more substantial. Through youth self-reports, a comprehensive understanding of the effect of socialization behavior and parent-child interaction could be measured as these might provide the receiver of the parenting practice's perspective. They could also be an alternative to decrease socially desirable answers and memory bias from parent-self reports and help in addressing the difference in the perspective of the parent and youth during family therapies. The youth/child self-reports can also provide a personal experience of their parent’s behavior that helps identify the areas for intervention and improve parent-child interaction [11].

The present study

The theory of parental socialization of emotions has been explained differently by different researchers through either a combination of parental meta-emotion philosophy with the theory of stress and coping on one hand and the social learning mechanism, i.e., child’s expression and regulation of emotions, parent’s reaction to their child’s manifestation of emotions, and discussion and coaching of emotions of the child, on the other [3,5]. This study aims to construct a scale that can illustrate the broad construct of parental socialization of emotions in a compact and short frame, making it easy to utilize for theoretical testing and aiding research to understand the phenomenon. The tool construction has taken into factor core components used to indicate the process of parental-meta emotion philosophy, i.e., Awareness, Acceptance, and Coaching [5]. The dimensions of Awareness, Acceptance, and Coaching have already been successfully employed in the PMEI, which is used to explore parents’ thoughts and feelings about the emotions they experience and their child’s. These dimensions lie close to the construct of parental socialization of emotions, whose scale that is being constructed is intended to measure. The scale is purposefully designed for adolescents and young adults to understand their perception and experience of the parental socialization process.

The predominant research and scales available indicate the socialization of negative emotions with a reason the expression of negative emotions is viewed as difficult to regulate with visible behavioral and emotional problems, which is considered hard to deal with. The negative emotions also surface the concept of negative urgency. Although related to impulsivity, it also aptly connects with emotional dysregulation due to negative emotions. Negative urgency is defined as a rash or risk-taking behavior as a consequence of the experience of negative emotions, such as anger or sadness. A suitable method of parental socialization of emotions aids with expressing these emotions in a healthier way. However, the aspect of positive urgency is frequently overlooked, which is explained as risk-taking behavior, such as gambling and substance overuse as a consequence of positive emotional experience. Although negative and positive emotions are thickly related to impulsivity, they also stem from emotional dysregulation, which can be influenced by poor parental socialization of emotions, leading to features of borderline personality [12]. Inadequate parental socialization of positive emotions can lead to the display of those positive emotions in inappropriate situations, failing the child to train to sense the environmental context and address in a healthier manner [13]. Hence, this scale also takes into consideration both negative and positive emotions in the exploration of parental socialization of emotions.

## Materials and methods

The aim of the study is to develop a retrospective tool that predominantly includes the process of perceived parental socialization of emotions (PPSE) among adolescents and young adults. The segment of methodology strives to elucidate the intricate measures undertaken toward the execution of this study.

The research design used to construct, validate, and measure parental socialization of emotions was a cross-sectional research design. This is a type of research design used to study participants from a spectrum of age groups at the same time and are selected based on exclusion and inclusion criteria, which is a swifter and more cost-efficient manner to conduct a study [14].

Participants, sampling, and inclusion and exclusion criteria

The target sample was adolescents from the ages 13 years to 18 years and young adults from 19 years to 28 years inclusive of males and females. The sample was collected from various schools and colleges across Delhi and Gwalior including participants from both urban and sub-urban India. Centre For Research (Research Conduct and Ethics Committee) issued approval (ref. no. RCEC/00273/01/22). The sampling method used was purposive sampling with clear inclusive and exclusive criteria to minimize sampling error. The inclusive and exclusive criteria are mentioned as follows.

Inclusion Criteria

Participants were from the ages of 13 to 19 years (adolescents) and 20 to 28 years (young adults), both male and female, with knowledge of English, and scoring below 24 in the Depression Anxiety Stress Scales for Youth (DASS-Y) (see Appendix A) [15].

Exclusion Criteria

Individuals having a history of any significant physical illness, a history of significant psychiatric illness, organicity or any other medical issues, and intellectual disability were excluded.

Process for tool development and data collection

The tool was constructed in four phases to assure the thoroughness of the scale with regard to its construct and content validity. The fifth phase involved the process of data collection and analysis. The phases are described as follows:

Phase 1

A robust review process was done for which the database used was Scopus, which included studies from the journals of *Springer*, *PubMed*, *Elsevier*, *BioMed Central Ltd*, *Wiley*, *Mosby*, *Sage Publications*, *Blackwell Publishing*, *Emerald*, *Frontiers Media SA*, *Routledge*, *American Psychological Association*, *Oxford University Press*, *Cambridge University Press*, and *Taylor and Francis*. A consolidated 509 research papers were extracted and studied to note various scales available and being utilized to explore the concept of parental socialization of emotions. An array of scales, both parent report and child or adolescent or young adult report scales, were shortlisted to be comprehensively inspected. Each tool was analyzed, and their gaps were duly noted. Furthermore, the items of the scales were perused to understand the characteristics that could be applied to the tool being constructed and the facets that could not be applicable to the cultural norms, e.g., the CCNES had an item about dating that would not be well received in all cultures. Based on the exploration, three domains were selected that integrated the process of parental socialization of emotions to be measured in a compact form. The domains were proposed by Katz [5], which are Awareness, Acceptance, and Coaching, addressing the totality of parental socialization of emotions.

Phase 2

This phase involved generating an item pool for three dimensions, namely, Awareness, Acceptance, and Coaching. The goal is to create a 45-item tool in which each dimension would comprise 15 items. In order to create a strong tool, more than threefold items were generated to the intended 45 items so that during the process of content validity, inter-rater validity, and factor analysis items could be sieved to retain the best 45 items optimally measuring the construct. A total of 160 items were generated for the same. The items comprised statements addressing both positive and negative emotions. A few items of the scale were formulated with the intent of reverse scoring to rule out the data of participants that might randomly address the scale; these were item numbers 36, 37, 51, 78, 81, 83, and 90 prior to the random shuffling of the items for the final administration. 

Phase 3

After the creation of the item pool, content validity needed to be established. For the same, the first round of peer review was conducted by two experts to check for grammatical mistakes and ascertain face validity. Corrections were made based on the feedback of peer review. For the second time, the items were sent to experts for review. Feedback from experts was taken into consideration, and due changes were made. A total of 144 items were retained and sent for the third round of expert analysis. Finally, the items were sent for content and construct validity to three experts who specialized in the domain of tool construction and parenting practices who further rated each item on a four options rating (1 = not relevant; 2 = drastic revision needed to establish relevance; 3 = relevant but need minor alterations, 4 = very relevant) [16,17]. After the third round of expert review, a total of 131 items were retained for further analysis.

Phase 4

On the completion of content validity, the items were shuffled based on computer-generated random numbers from 1 to 131 to have a mixed and unbiased set of items for better reliability. Finally, data were collected for the 131 items. The items were to be rated on a Likert rating scale ranging from the option of strongly disagree = 1, disagree = 2, neither agree nor disagree = 3, agree = 4, and strongly agree = 5. The participants had to choose the option closest to how they perceived and experienced their interactions with their parents related to emotions. The scoring of the scale was done as indicated in the Likert rating; however, the scoring for reverse items 36, 37, 51, 78, 81, 83, and 90, which became item numbers 8, 24, 25, 85, 109, 111, and 143 post shuffling, was done in the opposite manner, i.e., strongly disagree = 5, disagree = 4, neither agree nor disagree = 3, agree = 2, and strongly agree = 1. 

Phase 5

Various schools and colleges were approached, and permission from the concerned authorities was sorted. Permission from parents was also sorted through the school for the conduct of data collection. The consent of participants was received through manual signatures on consent forms provided through hard copies (see Appendix B). Data were collected from 1,200 adolescents and young adults aged between 13 and 28 years. Further processing of data was done by eliminating false data or incomplete questionnaires and participants attaining a score above 24 in the DASS-Y [15]. After the cleaning of data, 761 data were retained and uploaded on an Excel sheet (Microsoft Corporation, USA) for further analysis. The data were then analyzed using exploratory factor analysis (EFA) with the help of Analysis of Moment Structures (AMOS), which is an extension of the Statistical Package for the Social Sciences (SPSS) [18], which is mainly useful for social science research.

## Results

The tool construction involved the process of establishing content validity, analysis of reliability, and EFA. The details of the analysis are mentioned in this section.

Content validity

As a prerequisite to data collection, the process of establishing content validity was vital to understand that the items of the scale described well the phenomenon it targeted to measure. For establishing content validity, two content validity aspects were taken into consideration, namely, the Item Content Validity Index (I-CVI) and Scale Content Validity Index (S-CVI). To calculate the CVI, certain rules were taken into consideration from prominent studies that were easy to follow [16,17,19]. To calculate the CVI, experts were approached with the benchmark that they were either experienced in tool construction or expert in the area of parenting. A minimum of three experts and a maximum of 10 experts needed to be taken into consideration for the assessment of content validity [16]. For this study, three experts were chosen, two of whom were experts in tool construction and one was knowledgeable in the domain of parenting. The experts had to rate each item based on a four-option ordinal rating scale: 1 being not recommended and 4 being highly recommended [19]. If the raters are less than five, then the I-CVI is calculated by reducing the four-point rating to a dichotomous rating; that is, the rating 1-3 (not recommended) is represented as 0 and the rating 4 (recommended) is represented as 1; inferring that all raters must give a rating of 4 for it to be considered a holding a good I-CVI. The I-CVI was calculated as the number of agreements on an item divided by the number of raters present. There have been several debates over the calculation of the S-CVI with the struggle to substantiate the optimal manner to calculate the same. Two of the three best and easiest ways to calculate the S-CVI were used in the current study [19]. The first S-CVI is calculated by applying the average of all the I-CVI of all items of the scale, which provided an S-CVI of 0.96. The second S-CVI was determined by calculating the average of the I-CVI by each rater, then summing the average I-CVI by each rater, and dividing by the number of raters. This method is denoted as S-CVI/Ave, which produced an S-CVI/Ave of 0.96. S-CVI/Ave, a synonym of the average congruency percentage (ACP), which is the standard criteria to assess the acceptability of the S-CVI, must be above 0.90, and an ACP of 0.96 was calculated as an outcome [20].

Participants and sociodemographics

The study was conducted in various schools and various colleges in New Delhi and Gwalior, Madhya Pradesh. The data collected from New Delhi comprised of participants from urban areas with a middle socioeconomic background. The data collected from Gwalior, Madhya Pradesh, included participants from urban and semi-urban areas from the middle socioeconomic status. All the participants had a functional knowledge of English and could read the questionnaire without difficulty. The dominant data comprised of female participants from middle adolescence studying in middle or high school.

The details of sociodemographics are described in Table 1 (see Appendix C for the sociodemographic detail form). The age group of middle adolescence ranged from 13 years to 15 years; late adolescence ranged from 16 years to 19 years; and young adults ranged from 20 years to 28 years. The participants belonging to middle school included ages 13 years to 15 years; participants studying in high school included ages 14 years to 18 years; and the participants pursuing under-graduation were mostly from ages 17 years to 23 years.

**Table 1:** Sociodemographic details of the participants

Variable	Number (n)	%
Gender		
Male	337	44.28
Female	424	55.71
Age		
Middle adolescence (13 years to 15 years)	304	39.94
Late adolescence (16 years to 19 years)	272	35.74
Young adults (20 years to 28 years)	185	24.31
School level		
Secondary level	250	32.85
High school	146	19.18
Undergraduate	314	41.26
Postgraduate	51	6.70

EFA

An integral part of tool construction is the EFA, which identifies latent factors and indicates their link to observed variables. In the present study, the EFA aided in eliciting the dominant factors, verifying the dimensions that were chosen, and establishing the items best suited for each factor.

The study ensured that the data were normally distributed and factorability of the data was confirmed. The Kaiser-Meyer-Olkin (KMO) test and Bartlett's test of sphericity were conducted to ascertain the factorability, identify the number of factors, and optimize the items in the tool. The KMO and Bartlett’s test of sphericity outputs are described in Table 2, where a KMO value of 0.5 and above is suggestive of the data being suitable for the purpose of factor analysis with Bartlett’s test of sphericity also indicating similar results.

**Table 2:** KMO and Bartlett's test of sphericity of the EFA output for the PPSE scale KMO: Kaiser–Meyer–Olkin, EFA: exploratory factor analysis, PPSE: Perceived Parental Socialization of Emotion

Kaiser–Meyer–Olkin (KMO) measure of sampling adequacy		0.97
Bartlett's test of sphericity	Approx. chi-square	45110.04
	Df	8515
	Sig.	0.00

The EFA also provided an output to which an eigenvalue above 3 was considered to assure the better quality of scale and its factors. A cumulative variance above 50% is considered good for tool construction. Table 3 shows the eigenvalue output of three factors above the eigenvalue of 3 indicating higher variance of the three factors.

**Table 3:** Eigenvalue of the EFA output for the PPSE scale Extraction method: principal component analysis EFA: exploratory factor analysis, PPSE: Perceived Parental Socialization of Emotion

Initial eigenvalues				Rotation sums of squared loadings		
Factor	Total	% of variance	Cumulative %	Total	% of variance	Cumulative %
1	5.347	7.57 %	55.62 %	22.890	17.473	17.473
2	4.247	3.24 %	56.43 %	16.376	12.501	29.974
3	3.103	2.37 %	57.22 %	4.205	3.210	33.184

Item-Total Statistics correlates each item of the scale to the total scale. A corrected-item correlation above 0.30 in each item is considered to have good efficacy. In the Item-Total Statistics output, items 37, 78, 81, and 90 have attained a corrected-item correlation below 0.30, suggesting a poor correlation of the item with the entire scale; however, due to a large number of items in the scale, deleting these items would have no changes in the reliability of the scale and a Cronbach’s alpha of 0.89 would be unchangeably maintained. The same has been described in Table 4. However, the above-mentioned items have been eliminated during the finalization of the tool for the confirmatory factor analysis (CFA) to maintain a better efficacy of the scale.

**Table 4:** Item-Total Statistics

Items	Scale mean if the item is deleted	Scale variance if the item is deleted	Corrected item-total correlation	Squared multiple correlation	Cronbach's alpha if the item is deleted
2	175.41	1016.09	0.65	0.50	0.89
3	175.56	1022.92	0.57	0.42	0.89
13	175.36	1029.46	0.55	0.43	0.89
15	175.36	1029.69	0.52	0.40	0.89
18	175.01	1036.74	0.47	0.35	0.89
24	175.24	1026.71	0.59	0.47	0.89
25	175.35	1024.86	0.60	0.43	0.89
26.	175.20	1017.13	0.64	0.52	0.89
28	175.27	1021.10	0.62	0.48	0.89
29	175.18	1020.24	0.65	0.48	0.89
31	175.33	1025.03	0.57	0.42	0.89
32	175.21	1016.41	0.63	0.50	0.89
33	175.40	1023.97	0.56	0.43	0.89
34	175.46	1023.68	0.52	0.35	0.89
35	175.21	1018.17	0.65	0.49	0.89
37	175.36	1047.61	0.23	0.27	0.89
38	175.36	1023.50	0.60	0.47	0.89
39	175.51	1019.32	0.58	0.49	0.89
46	175.32	1024.63	0.58	0.46	0.89
50	175.10	1036.22	0.46	0.30	0.89
57	174.85	1031.22	0.54	0.40	0.89
58	174.82	1032.45	0.50	0.46	0.89
62	174.73	1034.80	0.48	0.42	0.89
64	174.90	1035.12	0.47	0.33	0.89
66	174.84	1029.59	0.53	0.43	0.89
72	174.90	1028.92	0.56	0.44	0.89
73	175.41	1024.09	0.57	0.43	0.89
78	176.06	1059.85	0.08	0.36	0.89
81	175.72	1059.33	0.07	0.37	0.89
86	175.11	1021.87	0.64	0.48	0.89
90	175.60	1042.06	0.28	0.33	0.89
93	175.47	1016.46	0.67	0.54	0.89
94	175.46	1015.79	0.65	0.50	0.89
95	175.24	1022.60	0.62	0.46	0.89
96	175.31	1017.96	0.64	0.48	0.89
97	174.91	1031.22	0.51	0.44	0.89
99	174.71	1042.16	0.33	0.26	0.89
100	175.21	1031.13	0.52	0.41	0.89
104	175.31	1021.36	0.63	0.47	0.89
121	175.51	1022.05	0.59	0.47	0.89
123	175.24	1020.19	0.60	0.48	0.89
124	175.20	1025.94	0.62	0.49	0.89
126	175.45	1018.01	0.67	0.54	0.89
127	175.48	1014.41	0.65	0.51	0.89
128	175.40	1020.02	0.65	0.49	0.89
131	175.50	1021.48	0.63	0.50	0.89
134	175.06	1021.37	0.64	0.50	0.89
136	174.98	1027.36	0.58	0.44	0.89
138	175.13	1024.72	0.61	0.43	0.89
139	175.14	1016.52	0.67	0.53	0.89

The focus of this study was to reduce the large number of items to a compact set of factors along with establishing the dimensionality of the items that the tool contains. The extraction method of principal component analysis (PCA) was used, which justifies the rationale of reducing the dimensionality of the data through the identification of important variables and clustering them into components also known as factors. The rotation method used was varimax along with Kaiser normalization, where varimax aids in the simplification of the interpretation of factor solution, and Kaiser normalization helps the factor loadings to be comparable to other factors by normalizing before the rotation and de-normalizing after the rotation [21]. After the EFA output, the items with a factor loading above 0.50 were taken into account, and the items loading below that were rejected. Furthermore, the items loading at two or more factors were discarded, and the items loading at a particular factor were taken into consideration. The final output of EFA consequent to the deduction of items was 46 items, as shown in Table 5. The inter-factor correlation was calculated to establish a strong relationship among the dimensions chosen. The output (Table 6) established using Pearson correlation suggests a good correlation among the factors. Appendix D shows the complete Perceived Parental Socialization of Emotion (PPSE) scale.

**Table 5:** Exploratory factor analysis output for the PPSE scale *Note*. N = 761. The extraction method: principal component analysis with an orthogonal (varimax with Kaiser normalization) rotation. PPSE: Perceived Parental Socialization of Emotion

Awareness dimension		Loadings		
		Factor 1	Factor 2	Factor 3
2.	My parents would frequently have a conversation with me to know if I was joyful.	0.64		
3.	My parents took a keen interest in knowing if I was emotionally doing well.	0.65		
13.	If I behaved enthusiastically, my parents would know the reason for my enthusiasm without the need for explanation.	0.57		
15.	Whenever I had a sarcastic laugh, my parents could easily tell the reason behind my laugh.	0.51		
18.	Whenever I looked delighted over the weekends, my parents knew I was looking forward to enjoying my holidays.	0.52		
24.	If something exciting took place, my parents would have already predicted as to how I would be expressing my excitement.	0.56		
25.	Whenever I appeared agitated, my parents would enquire about my feelings out of concern.	0.55		
26.	Every time I felt dull, my parents would try to ask me as to what was making me feel that way.	0.59		
28.	Whenever I looked upset, my parents would be eager to know as to what was the reason for my emotional state.	0.56		
29.	Whenever I seemed disappointed over a failure, my parents would want to talk to me about my feelings.	0.53		
31.	Whenever I had a fight with my friends at school, my parents would know that something was upsetting me.	0.60		
32.	When I felt upset and tried to hide from my parents, they somehow sensed something was bothering me.	0.64		
33.	My parents were aware of the situations that made me uncomfortable without sharing with them.	0.60		
34.	My parents knew all my friends and could easily tell if there was any tension among us.	0.52		
35.	If I had a bad day at school my parents could easily recognize that something was bothering me.	0.57		
38.	Whenever I looked dejected over a task I found difficult, my parents would talk to me about that task even before I could share about it with them.	0.57		
39.	When I looked frustrated, my parents would know what was going on inside me.	0.68		
46.	Whenever I was upset, my parents knew how I would be dealing with my feelings.	0.55		
Acceptance imension		Loadings		
		Factor 1	Factor 2	Factor 3
50.	If I started laughing loudly over a situation, my parents described me as being in a good mood.		0.50	
57.	If anything exciting happened such as winning a competition, my parents would tell me that they are proud of me.		0.53	
58.	Whenever I joyfully shared about my small achievements, my parents would complement me.		0.54	
62.	If I was elated about doing well at school, my parents would respond by saying 'well done'.		0.53	
64.	Whenever I got excited about going for a school picnic, my parents would wish me 'have fun'.		0.52	
66.	I have noticed that my parents smiled more whenever I was happy.		0.55	
72.	Every time I showed my gratitude by doing something special for my parents, they would smile at me with appreciation.		0.59	
73.	Every time I felt sad due to which I was unusually quiet, my parents would share that I seemed low.		0.52	
86.	If I felt low about having a hard time at school, my parents would assure me that things would get better.		0.53	
94.	If I felt dejected over being wronged by someone, my parents would give me a hug so as to make me feel better.		0.62	
95.	Whenever I felt disheartened over something I couldn’t achieve, my parents would check on me with concern.		0.54	
96.	Every time I felt emotionally overwhelmed, my parents patted me on my back to let me know they were there.		0.58	
97.	Whenever I felt delighted over receiving an award, my parents would let me express my joy to them without restrictions.		0.64	
99.	I could laugh in a relaxed manner in front of my parents without the fear of being scolded.		0.54	
100.	Whenever I felt jolly about meeting my friends or extended family, I could freely express my joy before my parents without being belittled.		0.55	
Coaching dimension		Loadings		
		Factor 1	Factor 2	Factor 3
104.	If I felt cheerful, my parents and I would do the activities we enjoyed together while discussing my feelings.			0.545
121.	Whenever I had a day where I felt irritated and behaved likewise, my parents would address my emotions in an understanding way.			0.580
123.	Whenever I felt like crying, my parents would hear me out with concern instead of dismissing my feelings.			0.527
124.	When I felt overwhelmed my parents would acknowledge my emotions to help me.			0.536
126.	During a low phase, my parents talked about my emotions and validated them.			0.702
127.	Whenever I had an anger outburst, my parents would talk to me regarding what caused it.			0.682
128.	Whenever I had a day where I felt irritated and behaved likewise, my parents would discuss with me about it.			0.615
131.	My parents would often discuss about what was bothering me and how I could express if I had an angry fit.			0.689
134.	Whenever I made a mistake, my parents would talk to me as to what I had done wrong and how I could improve.			0.507
136.	If I was worried about choosing something important, my parents would comfort me and guide me to make the best decision.			0.511
138.	If I felt frustrated over making a choice, my parents helped me to see the pros and cons for the choices at hand.			0.507
139.	Whenever I felt demotivated my parents would remind me of my capabilities.			0.508
93.	Whenever I felt overwhelmed, my parents would take out time to be with me.			0.665

After establishing the factors, the inter-factor correlation was calculated so as to ascertain the strength of the relationship among the dimensions established. Table 6 indicates the relationship among the dimensions, which was established using Pearson correlation coefficient. The results suggest a strong correlation among the factors.

**Table 6:** Inter-factor correlation *p < .05. **p < .01.

Variables	M	SD	Awareness	Acceptance	Coaching
Awareness	65.30	13.39	-		
Acceptance	55.88	9.84	0.799**	-	
Coaching	58.08	11.05	0.882**	0.819**	-

Reliability of the tool

Cronbach’s alpha was used to check the internal consistency of the test. Although test-retest reliability is preferred by researchers, the same is difficult to establish with an attitude scale wherein the approach, mental state, and mood of the participants tend to change over days leaning toward altering the responses [22]. Cronbach’s alpha is well used along with the EFA to establish internal consistency. It is usually used after the application of the EFA to assess the internal consistency factors [23,24]. Cronbach’s alpha reliability is usually considered equivalent to the split-half reliability [25]. In the current study, Cronbach’s alpha was used to ascertain that each of the items in the respective factors measures the consistency and the latent construct of the scale details provided in Table 7. The output of Cronbach’s alpha in the domains Awareness, Acceptance, and Coaching is above 0.80, suggesting a very good reliability. Furthermore, the Cronbach’s alpha of the entire scale was noted to be 0.88 (Table 7), indicative of the good internal consistency of the scale [26].

**Table 7:** Cronbach’s alpha coefficient after EFA EFA: exploratory factor analysis

Subscales	Cronbach’s alpha coefficient after EFA
Awareness	0.89
Acceptance	0.84
Coaching	0.87
Scale total	0.88

## Discussion

Parental socialization of emotions is described as the parent-child relationship revolving around emotions containing the parent’s reaction to the emotions expressed by the child, the discussion between the parent and child regarding the emotions expressed, and the emotional response of the parents toward their child [3]. Parent-child interaction is crucial to emotional development later during adolescence [27]; however, parental socialization of emotions has an integral role in several aspects, such as emotional regulation, emotional climate, emotional expression, emotional understanding, social competence, and psychological adjustment [3,28,29]. Hence, it is essential to be able to assess the concept of parental socialization of emotions not just for further research but also for a better understanding of the participant’s perspective for therapeutic intervention. The concept was given by Eisenberg [3] as concise parental socialization of emotions to a more behavioral approach, describing it under one of the emotion-related socialization behaviors with three components: parental response to the child’s emotions, parental discussion, and parental expression of emotions. This study has considered the analogy given by Katz [5] who introduced parental socialization of emotion under the domain of parental meta-emotion philosophy and depicted the phenomenon stratified as Awareness (parents’ interest in their emotional experience, ability to identify their emotions, discern the reason for the emotions, knowledge as to how they might express, and the manner in which it can be addressed), Acceptance (parent’s understanding of the manner in which they perceive their child’s emotion and describe the emotions and the way in which they respond to the emotional experience both verbally and non-verbally), and Coaching (the extent to which the parents involve themselves in contributing toward the strategies for managing and regulating their emotional experience and expression with the belief that parents show confidence in the ability of their child to deal with their emotions rather than refuting or belittling their emotions). It also provides a step-by-step and holistic view of parental socialization of emotions. There are several scales measuring the phenomenon, but a scale with the framework addressed in this study with adolescents’ and young adults’ perspectives is necessary.

Awareness domain and parental socialization of emotion

The domain of Awareness seeks to measure the extent of knowledge the parents have about their child’s experience of emotions at any given moment. This requires the parents to also have emotional intelligence to a certain extent in order to be able to be aware of the changes in their child’s emotions. However, being aware of the child’s emotions is as integral of a dimension as Acceptance and Coaching since being mindful of their child’s emotions is the stepping stone to choosing to react in an informed manner. The Parental Meta Emotion Coding System (Manual: Hunter, Hessler, Katz, Hooven, & Mittman. Parental meta-emotion coding system: Revised Version; 2006) elaborately explains the domain of awareness as the parent’s ability to observe and decode the emotions their child is experiencing, taking consideration of the parent’s interest in the child’s emotional life, and knowing the exact emotions their child is experiencing along with the reason and right accurate anticipation of how their child would express the emotion. The ability to notice the slightest changes in the emotional atmosphere of the child along with the ability to distinguish among emotions is the core of the Awareness domain, which paves the way to the aspect if the parents may or may not accept the emotion felt by the child. This study considered the perspective of adolescents or young adults and the extent to which they thought their parents had awareness of the emotions the adolescents or young adults experienced.

Acceptance domain and parental socialization of emotion

The domain of Acceptance could be assessed both as a consequence of the domain of awareness and an independent domain. Awareness of the child’s emotional experience provides a choice for the parents to accept or dismiss the emotion expressed by the child. Acceptance in this study refers to the parent’s acknowledgment and regard toward the child’s emotional expression in an understanding manner along with perceiving the reason for the emotions experienced. Acceptance is a factor that not only acknowledges the child’s emotions but also receives the emotional experience in a manner without judgments or reprimanding. The EFA results aided in reducing the items of the dimension to 15 items. The detail of the domain further addresses the extent to which the parent feels comfortable with their child’s emotions and their expression of those emotions, empathizing with the emotions that the child is feeling, normalizing the experience of the emotions felt, valuing talking about those emotions, concerns regarding the appropriateness of expressing those emotions, and choosing to get involved when the child is soothed when negative emotions need to be addressed (Manual: Hunter, Hessler, Katz, Hooven, & Mittman. Parental meta-emotion coding system: Revised Version; 2006). The retrospective understanding of the adolescents or young adults regarding the aspect of acceptance will also aid us in understanding their relationship with their parents, which might help during the therapeutic process.

Coaching domain and parental socialization of emotion

Coaching is the final aspect of parental socialization of emotions, which is also perceived as the foundation of the mechanism that is proposed [2,3]. Coaching enables parents to engage in conversation with their child regarding the child’s emotional experience and the parent’s. It gives an opportunity to create a parent-child bond, guiding and sharing experiences. The conduction of the EFA aided in the reduction of items to 14 items in the domain of coaching, which would enable further data analysis to determine the CFA. This dimension is clearly described in the aspect of the PMEI, where the scale of coaching includes the parent’s address of situations that arise from the experience of emotions, having a conversation with the child that is centered around the emotion-related situation along with the emotions experienced, engaging with the child to teach regarding healthy expression and regulation of emotions and being a source of comfort to the child [30]. The dimension does not just include the domain of coaching but has also integrated the subscales of the Child Behavioral Strategies from the Parental Meta Emotion Coding System (Manual: Hunter, Hessler, Katz, Hooven, & Mittman. Parental meta-emotion coding system: Revised Version; 2006). In the current scale being constructed, the Coaching domain also involves the approach of physical and verbal comfort, mental strategies, and restraining. This is done to include the essential aspects of the domain of Coaching including problem-focused coping, emotion-focused coping, and knowing when to use which strategy.

Comparison with existing tools

The current tool construction has been intricately developed keeping in mind the literature provided by Katz [5] proposing the dimension of parental socialization of emotions. The dimensions of the tool proposed by Fabes [6] mainly focus on the coaching aspect of parental socialization of emotion, also highlighted in the study that proposed their tool [30]. Another tool is the Emotion as a Child scale (Manuscript: Magai. Emotions as a child; 1996) [31,32], which is a self-report scale developed to assess the socialization of emotions by parents from the view of adolescents and emerging adults. The scale was developed to address four emotions, namely, anger or fear, sadness, anxiety, and shame, which are taken into consideration to assess five distinct strategies based on Malatesta-Magai’s model, namely, reward, override, punish, neglect, and magnify [33], which were considered as subscales. The Affective Meaning Questionnaire [34] was constructed to understand the parental control behaviors and their affective meaning aimed to be filled by young adults. It is comprised of four non-supportive parental socialization of emotion behaviors, i.e., punished, teased, maximized, and ignored, with three subscales depicting three different emotions of love, hurt, and shame. Although the scale is well intended to explore the young adult’s perspective, the tool only focuses on non-supportive parenting practices with an ambiguous structure that is being modified frequently. The Socialization of Emotion Scale (SES) [35] comprises three subscales with 12 items in each subscale assigned as parental distress reactions (parent distress), parental punitive reactions (parent punish), and parental minimization reactions (parent minimize). The scale aids in exploring only the perceived reaction of parents to the emotions expressed by the child. However, the process of parental socialization of emotions is more advanced where the perception of the emotions expressed, awareness of the emotions, acceptance or rejections of the emotions, and coaching of the emotions have not been taken into consideration, which represents the entirety of parental socialization of emotions. This scale has been specially developed to address all the gaps, such as measuring the process of parental socialization of emotions and establishing the domains that have been discussed since the inception of the phenomenon of parental socialization of emotions and the target population of adolescents and young adults.

Limitations and future research directions

The scale is yet to undergo the process of CFA that will further consolidate the scale for standardization. Although there are several scales for parental socialization of emotions, scales with a consolidated functional definition along with the domains proposed in the study are yet to be generated; hence, criterion validity could be measured along with the outcome scales of emotional competence (awareness, expressivity, and regulation).

## Conclusions

The study was conducted with the purpose of establishing a tool that could be built on a well-defined concept of parental socialization of emotions with precise dimensions taking into consideration both positive and negative emotional experiences. The EFA aided in establishing the three dimensions and reducing the items to 46 items, which would be used further to collect data for CFA. The Cronbach's alpha was used for establishing the reliability of each dimension and the entire scale after the EFA was above 0.80, indicating good internal consistency of the scale. The scale is intricately designed to explore the perspective of adolescents and young adults that might help in understanding and addressing past and present parent-child relationships. Understanding of emotional and social competence along with the contribution of parenting in its development might aid the therapist and client to navigate through the therapeutic process in a clearer way. The scale could also contribute toward exploring the pattern of parental socialization of emotions specific to communities, which would in turn enable in spreading of awareness regarding healthy parenting practices.

## Appendices

Appendix A

**Table 8:** Depression Anxiety Stress Scale - Y

DASS-Y		Name:	Age:	Date:				
We would like to find out how you have been feeling in THE PAST WEEK. There are some sentences below. Please circle the number that best shows how TRUE each sentence was of you during the past week. There are no right or wrong answers.								
If the statement was NOT TRUE of you (in the past week), circle 0								
If the statement was A LITTLE TRUE of you, circle 1.								
If the statement was FAIRLY TRUE of you, circle 2.								
If the statement was VERY TRUE of you, circle 3.								
1	I got upset about little things.				0	1	2	3
2	I felt dizzy, like I was about to faint.				0	1	2	3
3	I did not enjoy anything.				0	1	2	3
4	I had trouble breathing (e.g. fast breathing), even though I wasn't exercising and I was not sick.				0	1	2	3
5	I hated my life.				0	1	2	3
6	I found myself over-reacting to situations.				0	1	2	3
7	My hands felt shaky.				0	1	2	3
8	I was stressing about lots of things.				0	1	2	3
9	I felt terrified.				0	1	2	3
10	There was nothing nice I could look forward to.				0	1	2	3
11	I was easily irritated.				0	1	2	3
12	I found it difficult to relax.				0	1	2	3
13	I could not stop feeling sad.				0	1	2	3
14	I got annoyed when people interrupted me.				0	1	2	3
15	I felt like I was about to panic.				0	1	2	3
16	I hated myself.				0	1	2	3
17	I felt like I was no good.				0	1	2	3
18	I was easily annoyed.				0	1	2	3
19	I could feel my heart beating really fast, even though I hadn't done any hard exercise.				0	1	2	3
20	I felt scared for no good reason.				0	1	2	3
21	I felt that life was terrible.				0	1	2	3

Appendix B

**Table 9:** Consent form

I understand that this research aims at exploring the role parenting plays in emotional-social processing through tool construction where I am asked to fill the scale in retrospect to my parent’s role towards my emotional experience and expression. I voluntarily agree to participate in this research study as well as understand and acknowledge that even if I agree to participate now, I can withdraw at any time without any consequences of any kind. I understand that I can withdraw permission to use data from my interview within two weeks after the interview, in which case the material will be deleted. I have had the purpose and nature of the study explained to me in writing and I have had the opportunity to ask questions about the study. I accept that I will not benefit directly from participating in this research. I understand that all information I provide for this study will be treated confidentially.	
NAME:	
DATE:	SIGN:

Appendix C

**Table 10:** Sociodemographic detail form

I, Grace Sharon Joyce am pursuing my PhD from CHRIST (Deemed to be University), Delhi NCR, and as part of my PhD thesis, I am studying to explore parent's responses and interaction to their child's emotional expression, which is also known as Parental Socialization of Emotion, to which I am constructing a tool. It would be helpful if you could contribute to this study. Your personal results will be kept completely confidential, and if you would like to know your own individual results and their interpretation, I would be happy to share them with you through email ID grace.joyce@res.christuniversity.in.						
While filling out this form, you are requested to be as honest as possible. Also, there are no right or wrong answers.						
1. E-mail ID:						
2. Name:		3. Age:			4. Gender:	
5. Education qualification:						
6. Profession:						
7. Residence:						
8. Father’s profession:						
9. Level of education your father completed [Kindly choose (√) from the options provided below]:						
a. Completed 8th Standard						
b. Completed 10th Standard						
c. Completed 12th Standard						
d. Diploma or Skill School						
e. Bachelor’s Degree						
f. Master’s Degree						
g. M.Phil./Ph.D. or Higher						
h. Other Professional Degree (e.g MBBS, MBA. M.Tech, CA, CS)						
10. Mother’s Profession:						
11. Level of education your mother completed: [Kindly choose (√) from the options provided below]:						
a. Completed 8th Standard						
b. Completed 10th Standard						
c. Completed 12th Standard						
d. Diploma or Skill School						
e. Bachelor’s Degree						
f. Master’s Degree						
g. M.Phil./Ph.D. or Higher						
h. Other professional degrees (e.g., MBBS, MBA. M.Tech, CA, and CS)						
12. Who were your primary guardians while growing up (i.e., ages 0-18 years)? [Kindly choose (√) from the options provided below]:						
a. Mother & Father	b. Only Father		c. Only Mother	d. Relatives		e. Others
13. If your primary guardians were not your biological parents, kindly mention your primary guardians. _______________________________________________________.						
14. Did you live in the same house as your primary guardians while growing up (i.e., ages 0-18 years)? [Kindly choose (√) from the option provided below]:						
a. Yes			b. No			
15. If you did not live in the same house as your primary guardians while growing up, with whom did you live (i.e., ages 0-18 years)? _______________________________________________________.						

Appendix D

**Table 11:** Perceived Parental Socialization of Emotion (PPSE) scale

Instructions: Below are a few statements indicative of your experience during childhood. Against each of them are five options indicating the ratings as 'Strongly Disagree', 'Disagree', 'Neither Agree Nor Disagree', 'Agree' and 'Strongly Agree'. You are to choose the option closest to what you perceive for each sentence. There is no right and wrong answer and hence you are requested to attempt each item as honestly as possible.					
Statements	Strongly disagree	Disagree	Neither agree nor disagree	Agree	Strongly agree
My parents would light-heartedly tease me if I was being exceptionally thrilled about something that was my favorite.					
If I felt unwell before a major exam, my parents would be around me to hear me and take care of me.					
Whenever I happily shared with my parents about winning in a competition, my parents would clap for me.					
Whenever I felt scared my parents would try to help me find ways to gain more confidence.					
My parents would talk to me about situations that might bother me before they occurred.					
If I felt elated, my parents would also share how they used to express themselves at my age.					
Whenever I was in a bad mood over an argument with my siblings or friends, my parents claimed that I was upset.					
Whenever I expressed my anger, my parents called me dramatic.					
I could laugh in a relaxed manner in front of my parents without the fear of being scolded.					
Whenever I joyfully shared about my small achievements, my parents would compliment me.					
If I was elated about doing well at school, my parents would respond by saying 'well done'.					
If I was feeling discouraged over a difficult task, my parents would lend me a hand so as to motivate me.					
If I behaved overjoyed over a hobby I was pursuing, my parents complimented that I was doing a good thing.					
Whenever I had a day where I felt irritated and behaved likewise, my parents would address my emotions in an understanding way.					
Whenever I felt like crying, my parents would hear me out with concern instead of dismissing my feelings.					
If I had a day where I felt being on the edge, my parents would describe me as being nervous.					
If I failed at something for which I worked hard and felt demotivated about it, my parents would tell me that it was okay to feel that way.					
When I felt overwhelmed my parents would acknowledge my emotions to help me.					
If I felt agitated over tough homework, my parents would sit next to me as moral support.					
During a special occasion at home, I could be jovial in front of my parents without being hesitant about being described as immature.					
If I felt apprehensive, my parents would acknowledge it and let me know that they are there for me.					
My parents would explain that I was feeling sad, if I felt upset over losing my favorite object.					
Whenever I felt glad about completing a huge task without assistance, my parents would give me a pat on my back.					
My parents could not recognize if I was visibly sad and that I needed them.					
Whenever I shared something that was upsetting me, my parents thought I was seeking attention.					
If I contested in an activity and felt delighted about it, my parents could identify how I am feeling.					
My parents knew how I would respond if someone disturbed me when I didn’t want to be disturbed.					
My parents took a keen interest in knowing if I was doing well emotionally.					
My parents believed that I could handle my happiness without making any reckless decisions.					
When play time would be approaching my parents would know that I was in a joyous mood.					
Whenever I had exciting news to share, my parents would smilingly listen to me.					
If I was actively preparing before a special occasion, my parents would claim that I was behaving excitedly.					
If I was sad about a mistake I made, my parents would calmly help me understand the mistake.					
Whenever I felt ecstatic, my parents would say that I was being excited.					
If I felt frustrated when things did not go the way I expected, my parents described that I was feeling disappointed.					
Whenever I felt scared and told my parents about it, they would give me assurance that they are there for me.					
If I was feeling hurt over a friend being rude towards me, my parents responded by saying that my feelings were genuine.					
If I felt angry over something I did not like, my parents would trust me on dealing with my emotions by myself.					
Whenever I won a competition, my parents discussed with me on how I could be happy without getting overconfident.					
Whenever I beamed with pride over attaining a set goal, my parents shared that I seemed satisfied.					
Just before summer vacations, my parents would narrate as to what I would be doing during that time in my blissful mood.					
Every time I felt sad due to which I was unusually quiet, my parents would share that I seemed low.					
Every time I felt dull, my parents would try to ask me as to what was making me feel that way.					
If I was confused about choosing from multiple exciting choices, my parents trusted that I could choose the best option without being swayed by my excitement.					
If I felt overjoyed about meeting my favorite people during summer vacation, my parents would discuss as to how I could handle my excitement.					
If I seemed thrilled, my parents would ask about it.					
Whenever I hugged my parents out of joy, my parents would reciprocate my hugs.					
My parents knew all my friends and could easily tell if there was any tension among us.					
Whenever I felt overwhelmed, my parents would take out time to be with me.					
Whenever I had an anger outburst, my parents would talk to me regarding what caused it.					
If my parents felt I was being jolly they would discuss as to how to express my emotions in a healthy manner.					
Whenever I received a gift, my parents would immediately guess how I would act out of joy.					
When I felt upset and tried to hide my feelings from my parents, they somehow sensed something is bothering me.					
If I was upset over having to eat something I didn’t like, my parents would claim that I was being fussy.					
Before sharing good news, my parents would have guessed rightly as to how I would be reacting out of gladness.					
After a test at school, if I was cheerful, my parents would know that I had done well.					
Every time I felt emotionally overwhelmed, my parents patted me on my back to let me know they were there.					
My parents would frequently have a conversation with me to know if I was joyful.					
When I looked frustrated, my parents would know what was going on inside me.					
Whenever I felt pleasant, my parents could recognize the change in my emotions.					
Whenever I gave a mischievous smile, my parents could tell the mischief I was planning.					
If I made a silly joke, my parents would shake their heads and laugh at me.					
If I felt distressed during a difficult situation, my parents would ask me to do my best.					
Whenever I felt disheartened over something I couldn’t achieve, my parents would check on me with concern.					
If I was happy about winning a prize or doing well in academics, my parents would ask me as to how I would like to celebrate.					
My parents would take out time to discuss the fun things I was doing during school and play.					
My parents would often aptly describe how I would behave when I felt cheerful.					
My parents would often discuss about what was bothering me and how I could express if I had an angry fit.					
If I displayed my anger in a manner that was not acceptable, my parents would explain how I could have expressed my emotions differently.					
Whenever there was a fun event at school such as a picnic or sports day, my parents would take an interest in knowing whether I felt excited.					
If I felt embarrassed over a mistake, my parents would assert that it was valid to feel the way I did.					
Whenever I had a fight with my friends at school, my parents would know that something was upsetting me.					
Every time I cheerfully described my win in a game with my friends, my parents would also discuss how I could express my emotions without hurting my friend's feelings.					
Before a special day such as my birthday or the first day of my new class, my parents could accurately describe how I might react out of excitement.					
My parents knew how I would react to particular tense situations even before they occurred.					
During a low phase, my parents talked about my emotions and validated them.					
Every time I felt annoyed, my parents could tell what was bothering me just by the way I spoke.					
If I seemed delighted over a compliment given to me, my parents would take the initiative to know all about it.					
Whenever I felt overexcited about an outing, my parents would be confident that I could take care of myself.					
During a social gathering, if I felt embarrassed, my parents would know what exactly made me feel that way even before speaking to me.					
Whenever I felt agitated my parents knew I could handle my emotions well.					
Whenever I felt delighted over receiving an award, my parents would let me express my joy to them without restrictions.					
Whenever I laughed at a funny instance, my parents would join me and crack a few jokes as well.					
If I felt apprehensive before an exam, my parents would be interested in knowing as to what I was emotionally experiencing.					
If I was angry at my friends and tried to share with my parents, they would ignore and go about doing their work.					
Whenever I was excited, my parents would tell me that I was being goofy.					
Whenever I felt agitated my parents would know the cause without me expressing the reason.					
Whenever I felt jolly about meeting my friends or extended family, I could freely express my joy before my parents without being belittled.					
Whenever I looked delighted over the weekends, my parents knew that I was looking forward to enjoying my holidays.					
If I was forced to meet someone I wasn’t comfortable meeting, my parents knew how I would show my displeasure.					
Whenever I was happy, my parents encouraged me on how I could think before speaking in order not to offend anyone.					
During a tense situation, my parents would rather be there as a support than solve my problem themselves.					
My parents would light-heartedly joke with me if I was being excited for something I had been waiting for in a long time.					
If anything exciting happened such as winning a competition, my parents would tell me that they are proud of me.					
Whenever I appeared agitated, my parents would enquire about my feelings out of concern.					
Whenever I was jolly about buying a new object, my parents would also share about how they felt while buying something precious.					
I have noticed that my parents smiled more whenever I was happy.					
Whenever I seemed disappointed over a failure, my parents would want to talk to me about my feelings.					
Whenever I was excited over being given a big responsibility, my parents believed that I can cope on my own.					
Whenever I was upset, my parents knew how I would be dealing with my feelings.					
If I felt low about having a hard time at school, my parents would assure me that things would get better.					
When I seemed unusually happy, my parents could notice it with ease.					
My parents could easily identify my enthusiasm towards the things in which I was interested.					
If I appeared joyful, I could sit next to my parents and share about my mood while exchanging talks about our emotions.					
My parents were aware of the situations that made me uncomfortable without sharing with them.					
If I felt cheerful, my parents and I would do the activities we enjoyed together while discussing my feelings.					
Whenever I seemed scared, my parents would talk to me about my fears and encourage me to do my best.					
If I felt discouraged over scoring low on a test, my parents would talk to me about how I could build my resilience.					
Whenever I cried while being upset my parents would scold me for crying.					
Whenever I was refused something I really wanted, my parents would know how I would express my grumpiness.					
Whenever I was annoyed over something with which I disagreed, my parents would label me as being aggressive.					
Whenever I was angry over a fight with my friends, my parents would believe that I could sort out myself rather than intervening.					
If I was worried about choosing something important, my parents would comfort me and guide me to make the best decision.					
Every time I showed my gratitude by doing something special for my parents, they would smile at me with appreciation.					
Whenever I made a mistake, my parents would talk to me as to what I had done wrong and how I could improve.					
If I had a bad day at school my parents could easily recognize that something was bothering me.					
If I was angry my parents knew what I would do to express my anger.					
Whenever I felt demotivated my parents would remind me of my capabilities.					
If I felt dejected over being wronged by someone, my parents would give me a hug so as to make me feel better.					
If I was frightened about giving an exam, my parents would motivate me and tell me to give my best.					
If I felt frustrated over making a choice, my parents helped me to see the pros and cons of the choices at hand.					
If I was thrilled about going for an outing, I would not feel hesitant over expressing my emotions in front of my parents without being judged.					
My parents and I would often go out for dinner or lunch to cherish cheerful moments.					
Whenever I looked upset, my parents would be eager to know as to what was the reason for my emotional state.					
If I had learned a new game and was excited about it, my parents could tell why I seemed that way even before I explained the reason to them.					
Whenever I looked dejected over a task I found difficult, my parents would talk to me regarding that task even before I could share about it with them.					
If I cried when I got hurt, my parents would let me cry and make sure I was doing fine rather than scold me.					
My parents encouraged me to share if anything was bothering me rather than bottling it up.					
If I started laughing loudly over a situation, my parents described me as being in a good mood.					
If I behaved enthusiastically, my parents would know the reason for my enthusiasm without the need for explanation.					
Whenever I would behave in a childlike manner, my parents would describe me as being mischievous.					
Whenever I got excited about going for a school picnic, my parents would wish me 'have fun'.					
My parents would cheerfully laugh whenever I did something silly out of excitement.					
Whenever I was overjoyed about going out with friends, my parents knew that I could keep my emotions in check without being impulsive.					
I could never hide my excitement from my parents as they would know something was different.					
My parents would describe me as being enthusiastic whenever I became happy over a gift I received.					
Whenever I had a sarcastic laugh my parents could easily tell the reason behind my laugh.					
If something exciting took place, my parents would have already predicted as to how I would be expressing my excitement.					
For a school event, if I am elated about given an important role, my parents trusted that I'd be able to perform my role without being carried away by my emotions.					
Whenever I had a day where I felt irritated and behaved likewise, my parents would discuss with me about it.					
If I was being grumpy, my parents would describe that I was expressing my anger.					
Whenever I got excited about watching my favorite television show or movie, I could show my emotions in the presence of my parents without the worry of being embarrassed.					
If I was happy and would express it with my parents, they would scold me for being immature.					
Whenever I was excited about going out with my friends, my parents discussed with me as to how to carefully have fun without being carried away.					
